# Effects of the long-term storage of human fecal microbiota samples collected in RNAlater

**DOI:** 10.1038/s41598-018-36953-5

**Published:** 2019-01-24

**Authors:** Julien Tap, Stéphanie Cools-Portier, Sonia Pavan, Anne Druesne, Lena Öhman, Hans Törnblom, Magnus Simren, Muriel Derrien

**Affiliations:** 1 Danone Nutricia Research, Innovation, Science and Nutrition Department, RD 128 – Avenue de la Vauve, 91767 Palaiseau, France; 2 SPScienceCom, Paris, France; 3 Department of Immunology and Microbiology, Inst. of Biomedicine, University of Gothenburg, Gothenburg, Sweden; 4 Department of Internal Medicine and Clinical Nutrition, Inst. Of Medicine, University of Gothenburg, Gothenburg, Sweden; 5 Center for Functional Gastrointestinal and Motility Disorders, University of North Carolina at Chapel Hill, Chapel Hill, NC USA

## Abstract

The adequate storage of fecal samples from clinical trials is crucial if analyses are to be performed later and in long-term studies. However, it is unknown whether the composition of the microbiota is preserved during long-term stool storage (>1 year). We therefore evaluated the influence of long-term storage on the microbiota composition of human stool samples collected in RNAlater and stored for approximately five years at −80 °C. We compared storage effects on stool samples from 24 subjects with the effects of technical variation due to different sequencing runs and biological variation (intra- and inter-subject), in another 101 subjects, based on alpha-diversity, beta-diversity and taxonomic composition. We also evaluated the impact of initial alpha-diversity and fecal microbiota composition on beta-diversity instability upon storage. Overall, long-term stool storage at −80 °C had only limited effects on the microbiota composition of human feces. The magnitude of changes in alpha- and beta- diversity and taxonomic composition after long-term storage was similar to inter-sequencing variation and smaller than biological variation (both intra- and inter-subject). The likelihood of fecal samples being affected by long-term storage correlated with the initial relative abundance of some genera and tend to be affected by initial taxonomic richness.

## Introduction

The human gut microbiota, which may contain up to 10^14^ bacterial cells, is likely to play a major role in health and disease, influencing host physiology and the maintenance of immune homeostasis^1,2^. Associations between changes in gut microbiota composition and metabolic disease also suggest that gut microbes may modulate host metabolic function^3,4^.

A large number of longitudinal and prospective studies characterizing gut microbiota composition have been published in recent years^5–8^. Most of these studies were based on the collection of fecal samples, the most accessible material for studies of colonic microbial populations. The appropriate handling and storage of stools from clinical studies are crucial to prevent changes in microbiota composition, resulting in misinterpretation due to technical variations^9^. Meta-analyses and research studies comparing metagenomic or 16S rRNA gene sequencing datasets with those of other human microbiome studies are widely used to investigate the role of the gut microbiota or of specific species in health and disease^10–12^. The results generated may be influenced by technical variations^13,14^, including sample collection method, storage processes, DNA extraction method, choice of primers for the 16S rRNA gene, sequencing method, and bioinformatic tools. The technical variability between studies remains much smaller than the inter-individual variability, but may nevertheless mask subtle, meaningful, changes^15,16^.

Several studies have investigated the effects of different stool collection procedures and short periods of storage on microbiota composition, through comparisons with the gold standard technique of immediately freezing the fecal samples and storing them at −80 °C^17^. The main objective of these studies was to mimic the conditions of clinical studies, with collection of the stool sample at home followed by its transportation to the laboratory, to identify the most reliable methods for use in clinical studies. However, different collection procedures are still used, with room temperature “laboratory kits” (e.g. Fecal Occult Blood Test cards, Whatman FTA cards), home refrigeration or freezing, or the use of stabilization solutions kept at room temperature, 4 °C or −20 °C. Studies of the effects of long-term storage are also of interest because microbiota composition is investigated over long periods (years) in many clinical trials, and new analyses may be required after several years. Only a few studies have explored the impact of the long-term storage (>1 year) of fecal samples at −20 °C or −80 °C. They mostly found no significant difference between handling and storage methods, but minor differences were detected in studies involving more detailed analyses (specific genera)^18^. These changes were always smaller than the inter-subject variability. However, no study has explored intra-subject variability. One major challenge in studies of the long-term stability of DNA or stool samples relates to the rapid development and use of new sequencing technologies in microbiome studies. For analyses of stability over time, the same analytical approaches should be used throughout the study, as the sequencing platform and analytical software are technical sources of variability^13,16,19^.

In this study, fecal material from two cohorts of 24 and 101 human subjects was collected in RNAlater, and used to assess technical and biological variability in identical analytic approaches. The aliquoted stool samples were stored at −80 °C for five years. Alpha-diversity, beta-diversity and taxonomic composition were determined, with the aim of evaluating the effects of storage and comparing these effects with those of technical and biological variability (intra- and inter-subject variability).

## Materials and Methods

### Study design

**Figure 1:**
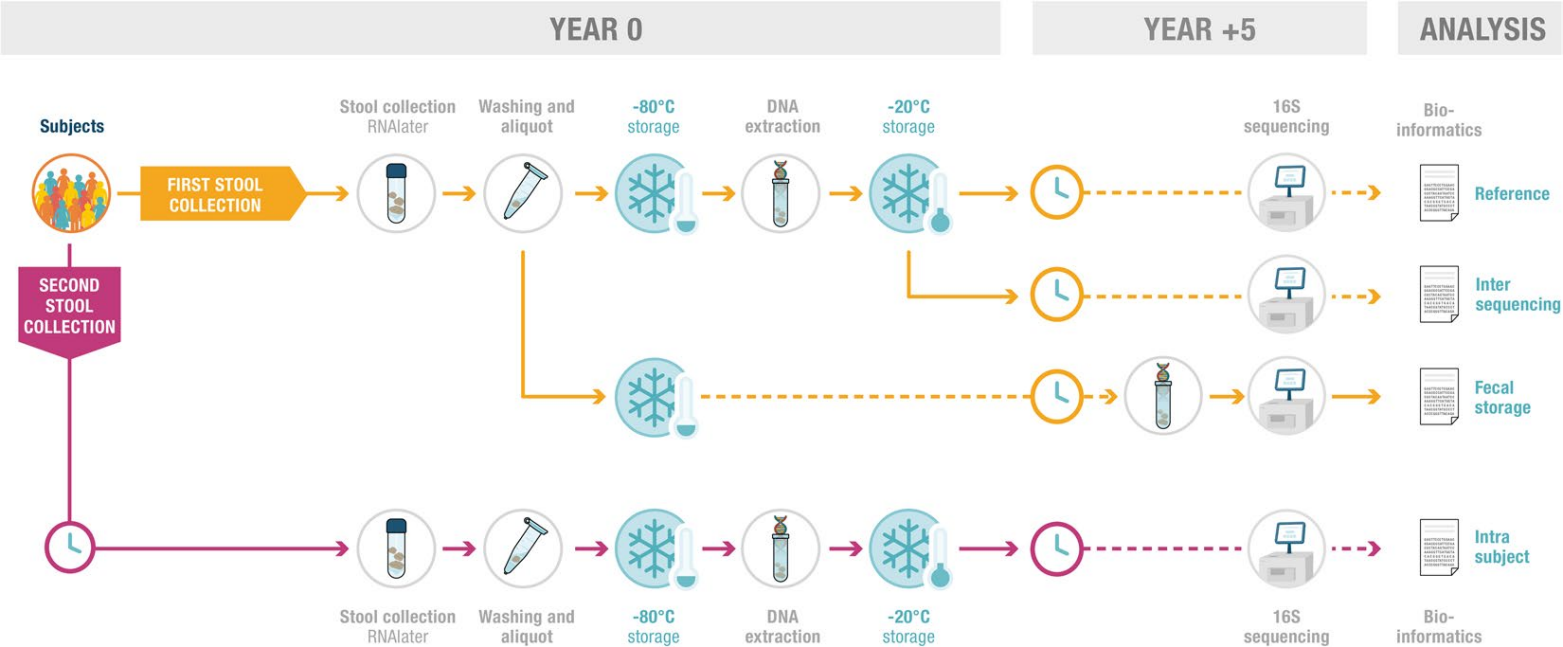
Study design. Fecal samples were collected from a total of 125 subjects (24 healthy adults and 101 IBS patients, see Supplementary Table S1). Samples were collected by the subjects at home, in RNAlater, and were then processed at the laboratory for dry storage at −80 °C. For each sample, one aliquot was extracted and sequenced to constitute the “reference” (year 0). Extracted DNA from 19 subjects was sequenced a second time, to study technical variability between two sequencing runs (“inter-sequencing runs”). One fecal aliquot from each of 24 subjects was used for DNA extraction and sequencing after long-term (five years) storage at −80 °C. The dataset obtained was compared with the corresponding dataset for reference values (stored DNA resequenced at year 5), to study the effect of long-term storage (“fecal storage”). A second round of stool collection was performed for 93 subjects. This second sample was used for DNA extraction and sequencing in year 0, and the resulting dataset was compared with the reference samples from the same subjects to evaluate biological variability within subjects (“intra-subject”). Inter-subject analysis consisted of an analysis of differences between subjects at year 0. Design Crea Nostra

We studied 218 fecal samples, collected from two previous cohorts (125 subjects) (Fig. 1 and Supplementary Table S1). The first cohort consisted of 24 healthy adult women from a study by Tillisch *et al*.^20^. Each subject provided one stool sample, which was collected in 2010. The effect of long-term stool storage was evaluated with the 24 samples from healthy subjects. For each fecal sample, one aliquot was extracted and sequenced to constitute the “reference” (year 0). One fecal aliquot from each of 24 subjects was used for DNA extraction and sequencing after long-term (five years) storage at −80 °C. The dataset obtained was compared with the corresponding dataset for reference values (stored DNA at −20 °C and resequenced at year 5), to study the effect of long-term storage (“fecal storage”).

The second cohort consisted of 101 subjects with irritable bowel syndrome (IBS) from a study by Tap *et al*.^21^. Each subject from this cohort provided two stool samples, collected between 2011 and 2013, with a mean of 26 ± 16 days between the two samples. We analyzed the variability between sequencing runs, by re-sequencing DNA from 19 randomly selected samples from the subjects with IBS from the reference set and determining inter-sequencing run variability (Study design shown in Fig. 1). Intra-subject variability was studied by comparing samples from the two rounds of collection for 93 IBS patients.

### Sample collection and handling

The subjects from the cohorts were asked to collect fecal samples immediately after defecation and to homogenize them with beads in RNAlater solution (Ambion, Austin, Texas) for storage at room temperature for 12 ± 5 days (Tillisch *et al*.^20^) or 11 ± 5 days (Tap *et al*.^21^). Each fecal suspension was adjusted to a final fecal dilution of 1:10 (wt/vol). We added 200 µl of the 10-fold dilution to 1 ml of phosphate-buffered saline (Invitrogen) and the samples were centrifuged for 5 min at 15,000 × *g*. The supernatant was discarded and the fecal pellet was stored at −80 °C until DNA extraction. DNA was extracted by repeated bead-beating in phenol/chloroform and stored at −20 °C, as previously described^22^. Briefly, fecal pellets were resuspended in a solution containing 450 μl of extraction buffer (100 mM Tris-HCl, 40 mM EDTA; pH 9.0) and 50 μl of 10% sodium dodecyl sulfate. Glass beads (0.1 mm diameter, 300 mg) were added to the suspension along with 500 μl of buffer-saturated phenol, and the mixture was vortexed vigorously for 30 s with a FastPrep FP 120 (BIO 101, Vista, California) at power 5.0. The suspension was then centrifuged at 14,000 × *g* for 5 minutes, and 400 μl of the supernatant was collected. Phenol-chloroform extractions were performed, and 250 μl of the supernatant was subjected to isopropanol precipitation. The DNA pellet obtained by centrifugation was suspended in 1 ml Tris-EDTA buffer and stored at −20 °C until use. All participants gave written, informed consent, and the study protocols were approved by the Regional Ethical Review Board in Gothenburg and Institutional Review Board at UCLA prior to the start of patient inclusion. All procedures complied with the principles of the Declaration of Helsinki.

### Microbiota profiling by 16S rRNA gene sequencing

We investigated microbial composition by 16S rRNA gene amplicon sequencing. Sequencing analysis was carried out at DNAVision SA (Gosselies, Belgium), on a 454 Life Sciences Genome Sequencer FLX instrument (Roche Applied Science, Vilvoorde, Belgium), with Titanium chemistry. The V5-V6 hypervariable 16S rRNA region was amplified with the specific primers 784F (5′- AGGATTAGATACCCTGGTA-3′) and 1061R (5′-CRRCACGAGCTGACGAC-3′)^23^. The forward primer contained the sequence of the titanium A adaptor and a unique barcode sequence. The data obtained were analyzed with the open-source software package “Quantitative Insights Into Microbial Ecology” (QIIME), v1.9^24^. Briefly, raw sequencing reads were filtered based on the following quality criteria: no mismatch with the primer sequences and barcode tags, no ambiguous bases (Ns), read-lengths between 200 base pairs (bp) and 1000 bp, mean quality score in a sliding window of 50 bp no lower than 25, with the exclusion of homopolymer runs of more than 6 nt in length. Sequences satisfying these quality filters were clustered into operational taxonomic units (OTUs) at the 97% identity level, with Vsearch^25^, and taxonomically assigned with the Silva 119 database^26^, resulting in 8700 ± 4072 sequences per sample.

### Quantitative PCR for total bacteria

Quantitative polymerase chain reaction (qPCR) was performed with a real-time polymerase chain reaction system (PRISM 7900HT; Applied Biosystems, Foster City, CA). All reactions were performed at least in duplicate. Comparison with other bacterial quantification methods was facilitated by converting the number of molecules detected (DNA) into cell equivalents. A culture of the reference bacterial strain indicated (grown in the appropriate medium and collected at stationary phase) was used to generate a standard curve of threshold cycle against bacterial cell number (determined microscopically with 4 = 6-diamidino-2-phenylindole staining from a dilution series of the reference strains). Standard curves of DNA from *Bifidobacterium longum* were plotted with 10^6^ to 10^10^ cells. Samples were analyzed in a 25-µl reaction mixture consisting of 12.5 µl SYBR Premix (50 mmol/L KCl, 20 mmol/L Tris-HCl, pH 8.4, 0.2 mmol/L deoxynucleoside triphosphate, 0.625 U Ta-KaRa Taq (Clonetech, Mountain View, CA), 3 mmol/L MgCl_2_, and 10 nmol/L fluorescein), 0.2 mol/L of each primer, and 5 µl of DNA. Serial dilutions (100–1000-fold) of extracted DNA were subjected to quantitative polymerase chain reaction with the Uni331F (5′-TCCTACGGGAGGCAGCAGT-3′) and Uni797R (5′-GGACTACCAGGGTATCTAATCCTGTT-3′) primers.

### Statistical analysis

Weighted and unweighted Unifrac, Bray-Curtis and Jensen-Shannon distance metrics^27,28^ were used to evaluate beta-diversity at the genus and OTU levels. Alpha-diversity metrics were evaluated by determining the number of OTUs, Shannon index, Chao index and Pielou index detected after sample read rarefaction (1000 sequences). For each subject, fecal storage effect, sequencing run effect (inter-sequencing run) and subject biological variability (intra-subject and inter-subject) were evaluated and compared with those of the corresponding reference samples, by calculating the number of species differences and determining beta-diversity metrics. Differences in diversity between the reference and follow-up samples were assessed with paired Wilcoxon tests, with *p* values < 0.05 considered significant. Microbiota sample instability was defined as the absolute difference in alpha- and beta-diversity in comparisons between the follow-up and reference samples. The proportion of each genus and the number of OTUs observed (richness) were also evaluated for the reference samples and their correlation with alpha- and beta-diversity instability during fecal storage was assessed by calculating Spearman’s rho. The association between the baseline mean relative abundance or prevalence of each genus and fecal sample stability was assessed previously described^8^. Differential analysis was done using DESeq2 library (version 1.14.1^29^, to assess the impact of fecal storage. on OTUs raw count matrix derived from Qiime biom file.

## Results

### Impact of long-term sample storage on fecal microbiota diversity and composition

**Figure 2:**
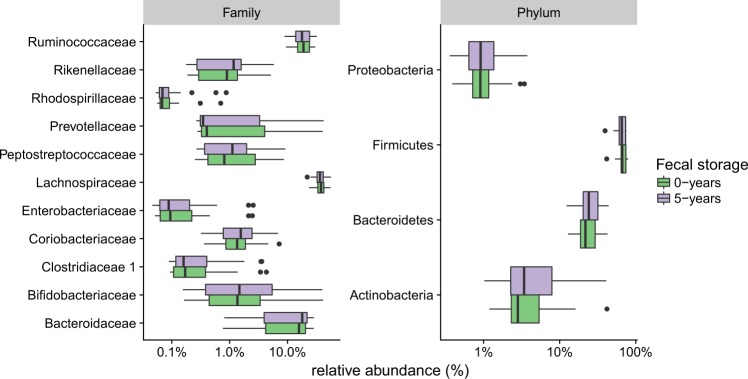
Boxplot of the relative abundance of phyla and families in fecal samples before and after long-term storage. Phyla or families accounting for more than 10% of the microbiota present in at least one sample are depicted. Microbial taxa were inferred from the SILVA database (version 119).

We first assessed whether the samples used to evaluate the impact of long-term storage were representative of the considerable variability normally observed in fecal microbiota studies, by determining the relative abundance of the dominant phylum and family taxa. A family or phylum was considered to be dominant if it accounted for more than 10% of the microbiota present in at least one sample. Four phyla (Actinobacteria, Bacteroidetes, Firmicutes and Proteobacteria) and 11 families were considered dominant (Fig. 2). Together, they accounted for at least 80% of the relative abundance of each sample. Their relative abundance in the samples studied varied substantially across samples (inter-individual variability). For example, the relative abundance of the Bacteroidetes phylum ranged from 13% to 43%, whereas that of the Prevotellaceae family ranged from 0.3% to 41%. We then investigated whether fecal storage for five years induced changes in the relative abundance of major phyla, families and genera (>5%). Microbiota profile was compared between fecal samples stored 5 years compared with the corresponding reference dataset to study the effect of long-term storage (“fecal storage”).

There was no significant difference between paired samples before and after storage at any of these taxonomic levels (paired Wilcoxon tests for these dominant taxa (Fig. 2 and Supplementary Fig. S1, p > 0.05). We extended the analysis using DESeq2, an approach specifically designed for RNA sequencing analysis and suitable for low number of subjects^30^. DESeq2 analysis did not show any taxonomical level (from phylum to OTU) affected by fecal storage.

**Figure 3:**
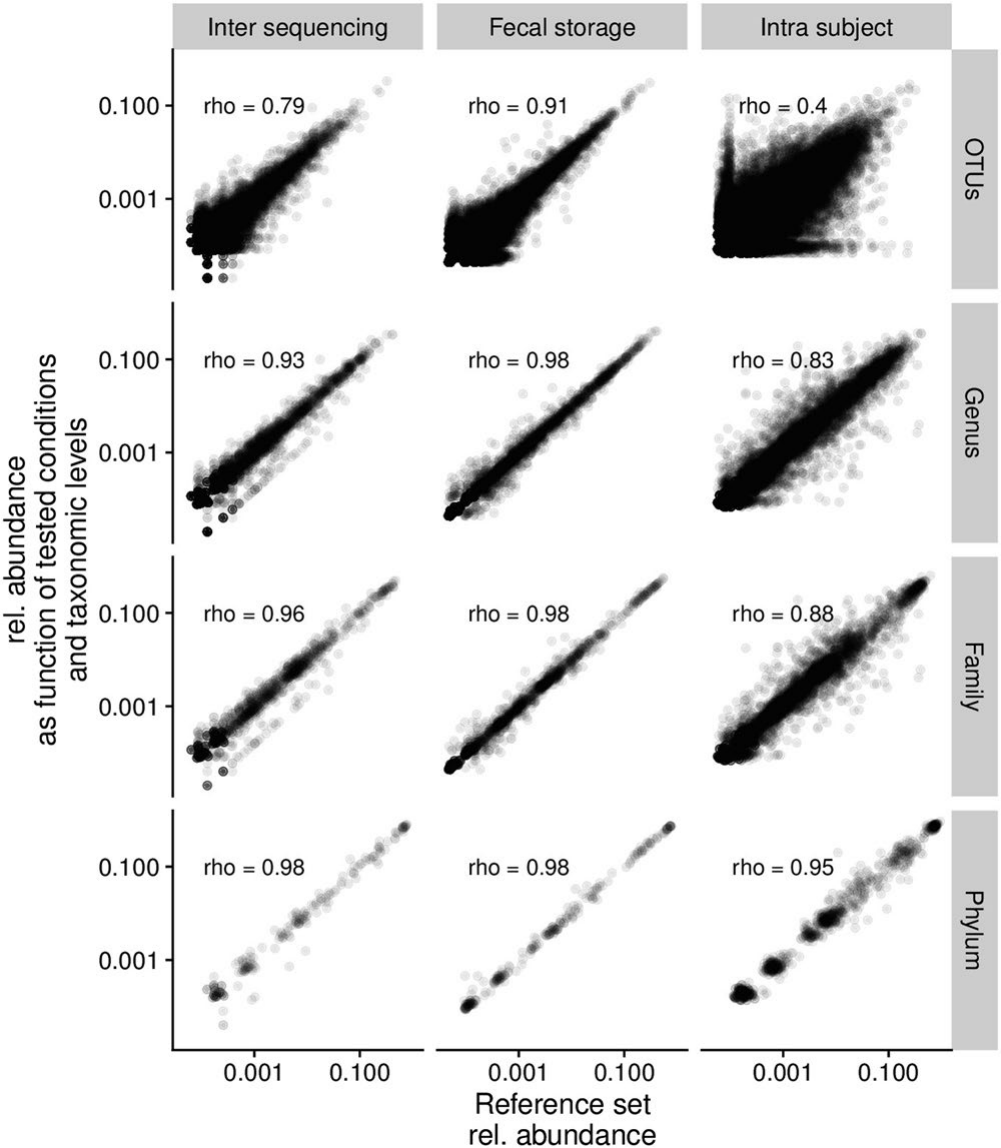
Scatterplots of the relative abundance of phyla, families, genera and OTUs. Relative abundances of microbial taxa in the conditions tested (inter-sequencing run, fecal storage, intra-subject) were plotted against the corresponding reference dataset. Spearman’s rho correlation coefficients for each set of conditions tested are shown on the graph.

We then compared relative abundance in each set of test conditions with that in the reference dataset, from phylum to OTU, in Spearman’s rank correlation test. For each taxonomic level, the technical variability between two sequencing runs was not different from the effect of long-term storage, and systematically smaller than the intra-subject variability (Fig. 3, *p* < 0.05). The correlation coefficient rho ranged from 0.95 to 0.98 at the phylum level, but was lower, at 0.83 to 0.93, at the genus level, depending on the conditions tested. At the OTU level, intra-subject variability was higher, whereas the technical variability between two sequencing runs and the effect of long-term storage remained small.

**Figure 4:**
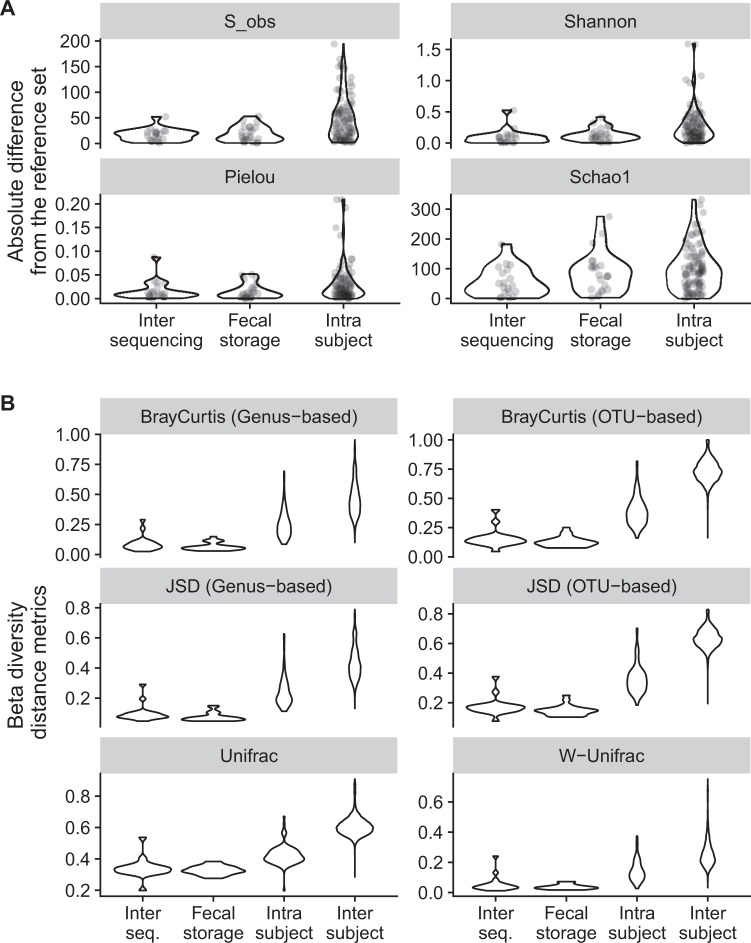
Violin plot of alpha- and beta-diversity metrics for each set of conditions tested, relative to the reference sample. (**A**) Alpha-diversity variation was evaluated as the positive absolute number of OTUs, Shannon, Chao, and Pielou indices, different from the corresponding reference. (**B**) Beta-diversity was assessed with Unifrac (unweighted and weighted), Bray-Curtis, and Jensen-Shannon metrics, at the genus and OTU levels.

Changes in alpha-diversity, assessed by determining the positive difference in the number of OTUs, Chao, Shannon and Pielou indices, between follow-up and reference samples, were larger within and between subjects (intra- and inter-subject respectively), than between sequencing runs and before and after long-term storage (Fig. 4a). Furthermore, similar results were obtained for changes in beta-diversity measured with the Unifrac, Bray-Curtis, and Jensen-Shannon metrics at the genus and OTU levels (Fig. 4b). The inter-sequencing run effect was not significantly different from the fecal storage effect. Inter-sequencing run and fecal storage effects were significantly smaller than the intra-subject variability (Wilcoxon test, *p* < 0.05). We also investigated whether the duration of storage in RNAlater at room temperature at the subject’s home affected stability, by assessing Bray-Curtis dissimilarity at genus level. No correlation was observed between Bray-Curtis index and the number of days (up to 23 days) of storage in RNAlater (Supplementary Fig. S2).

### Association between the baseline microbiota and the amplitude of change after long-term fecal storage

**Figure 5:**
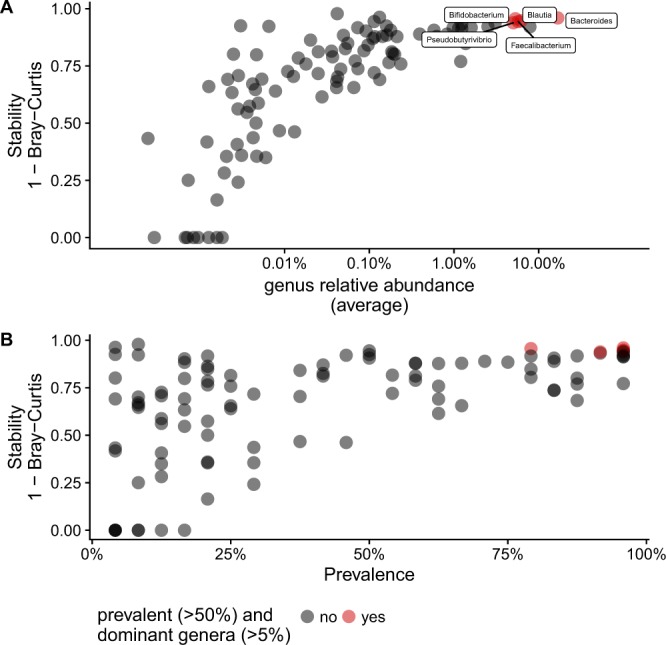
Stability of individual genera after storage for five years is correlated with mean baseline relative abundance and prevalence. Each point represents a genus (N = 99).

We then investigated whether some of the features of the initial gut microbiota, such as the relative abundance of genera and alpha-diversity, were associated with the amplitude of change (beta-diversity) after stool storage for five years. Using the complement of within-person BC dissimilarity score (1 – BC) as described by Mehta *et al*.^8^, over the various test conditions (versus its own reference set), we found that initial genus relative abundance were also strongly and positively correlated with long-term stability (Fig. 5a). Genera with prevalence above 50% were considered more likely to be stable (Chi square < 0.05) (Fig. 5b). For instance, the genera that were both prevalent and abundant included *Bacteroides, Bifidobacterium, Faecalibacterium, Pseudobutyrivibrio*, and *Blautia*. The genera with a low prevalence and abundance included *Methanobrevibacter* and were considered unstable (Supplementary Table S2). Higher baseline species richness (number of OTUs) tended to be associated with lower stability (rho = 0.37, p = 0.07). Total bacterial count, estimated by qPCR, was associated with species richness (rho = 0.71, *p* < 0.05) but not with beta-diversity (rho = −0.01, p = 0.96).

### Compilation of studies assessing microbiota stability based on collection and storage procedures

**Figure 6:**
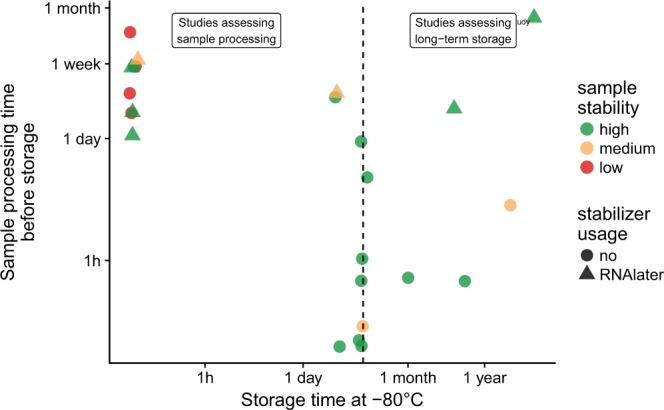
Graphical summary of studies assessing the impact of sample processing and storage on gut microbiota composition. The data presented were extracted from the studies reported in Additional file 1. Sample integrity was determined from the study conclusions, as listed in Additional file 1. For samples treated with a stabilizing buffer, we included in our analysis only those treated with RNAlater.

We assessed the extent to which the fecal microbiota was affected by collection processes and storage over time, by analyzing the outcomes described in 25 published studies focusing on both short- and long-term storage, and in this study (Supplementary Table S3). Most of the previous studies used samples from a small number of individuals (fewer than 10 subjects), whereas more recent studies enrolled larger numbers of subjects (*n* = 40). For each study, we retrieved the duration of sample collection and storage, and the stabilizer used. We simplified the analysis by including publications if fecal samples were processed without stabilizing buffer or with RNAlater, and if the duration of storage at −80 °C was indicated, which resulted in the selection of 11 studies (including this study). Based on the conclusion of each study, sample stability in each set of conditions tested was coded as “high”, “medium” or “low” (Fig. 6 and Table S3). Fecal sample stability was considered high if diversity (alpha and beta) and composition were not affected according to the conclusions of the publication concerned. Sample stability was most strongly affected by processing time (exceeding 24 hours) after collection and before long-term storage, in the absence of a stabilizer. Microbiota composition and diversity were found to be highly stable in all other conditions tested (Fig. 6). This suggests that the short-term processing of fecal samples (protocol and duration) is the most important factor affecting gut microbiota stability.

## Discussion

In this study, we evaluated the impact of long-term storage on the composition of fecal samples as determined by 16S rRNA gene sequencing. We analyzed human fecal samples collected in RNAlater and stored for long periods at −80 °C. The main findings were that long-term storage of stool samples at −80 °C had little effect on the integrity of the gut microbiota, although minor changes in the relative abundance of some species were correlated with initial microbiota composition.

We analyzed the effects of fecal storage on microbiota composition, comparing storage effects with the effects of technical variation due to different sequence runs and biological variation (intra- and inter-subject), to evaluate potential technical and biological bias. We found that long-term storage induced changes in the microbiota that were smaller than the intra-subject variability and similar to the inter-sequencing run effect. These results suggest that fecal samples collected in RNAlater can be stored for several years at −80 °C with no major deviation of their composition, provided that all sequencing runs on a given sample make use of the same sequencing platform and protocol. Larger differences were observed when different rounds of sampling were compared, reflecting the large intra-subject variability. It was possible to compare technical and biological variation (intra- and inter-subject), because we standardized our analysis and limited the introduction of technical bias by analyzing only samples sequenced with the same technical approaches (DNA extraction, primers, sequencing platform). The importance of these issues highlights the non-negligible obstacles likely to be faced when studies of this type are run over several years. Our study is consistent with other studies (Supplementary Table S3) showing that technical variability is lower than the inter-subject microbiota variability. Moreover, one of the strengths of our study is that it compares technical variability with intra-subject microbiota variability. This approach is highly pertinent, given the increasing number of longitudinal studies targeting the gut microbiota and the benefits of classifications based on a larger number of subjects^5–8^.

We further evaluated whether the residual variation of fecal samples induced by long-term fecal storage could be linked with initial microbiota composition. Higher initial richness tended to be associated with a moderately higher sensitivity to change during long-term storage. Bacterial counts were positively correlated with species richness, but not associated with fecal sample instability after long-term storage. Some changes in sample stability were observed during long-term storage, particularly for low prevalent and low abundant members. Our results are consistent with previous findings suggesting that storage effects may depend on microbiota composition^10,31^. Thus, depending on the baseline microbiota of the subject, long-term sample storage may affect the integrity of the fecal microbiota. These results also highlight the importance of studying sufficiently large numbers of subjects, differing in microbiota composition and variabilities of diversity/richness, for the detection of small differences and to ensure that the conclusions drawn are robust.

Most published studies have compared different approaches to stool collection and focused on the effects of short-term storage, from a few days to a few months (Fig. 6 and Supplementary Table S3). However, two studies extended the analysis to two years^32^ and 14 years of storage^10^. In the first study, Shaw *et al*., investigated the effect of long-term storage on infant stools, which have a less complex microbiota. They observed some changes in OTU relative abundance and composition during two years of storage^32^. However, diversity was more strongly affected by long-term storage in microbiota that were initially less complex. In the second study, Kia *et al*. compared the microbiota of 13 fecal samples stored at −20 °C immediately after collection and then freeze-dried with the results reported for the American Gut Project, for which similar DNA extraction and sequencing approaches were used. However, the samples were collected differently (room temperature versus direct freezing at −20 °C). No differences were observed at phylum level, but potential changes at lower taxonomic levels were not investigated^10^.

In our study, RNAlater was used as a stabilizer, to preserve fecal samples before their storage at −80 °C. Preservatives of this type are increasingly being used, due to their suitability for use at diverse storage temperatures, and for long time storage periods^33^. Stabilizers provide a good compromise between practicality for the self-collection of stools at home and reliability. However, their use has been controversial. Some studies have reported higher levels of microbiota variability, in terms of composition or relative abundance, for stools collected at home and insufficiently homogenized in RNAlater^34,35^, and for stools stored at room temperature in RNAlater for more than two weeks^36^. However, other studies have reported no major impact of RNAlater on the microbiota^37^. Furthermore, RNAlater has been shown to stabilize the microbiota more effectively than dry storage^15,38,39^. It is not yet possible to draw clear conclusions, due to several methodological differences, including the sequencing platforms and bioinformatics tools used, the duration of storage, baseline conditions or the absence of baseline, and the depth of taxonomic analysis. However, we cannot rule out the possibility that the use of RNAlater in our study introduced a bias, as it was not possible to compare the results obtained with the “gold standard” of immediate freezing of fecal samples. Thus, there might be an effect of RNAlater dependent on the initial microbiota of the subjects, or an impact of RNAlater on DNA extraction yield, as previously suggested^40^. However, we minimized this potential effect, by using a 1/10 dilution of the collected fecal samples in our study, to reduce RNAlater viscosity and to obtain higher bacterial recovery rates after centrifugation^41^.

In conclusion, the long-term storage of stool samples at −80 °C had little effect on the integrity of the gut microbiota, although minor changes in the relative abundance of some species correlated with initial microbiota composition were observed. However, these changes were always smaller than inter-individual and inter-sequencing run variability. These results also highlight the importance of performing studies with sufficiently large numbers of subjects, to make it possible to optimize the robustness of conclusions. We provide the first description of the effects of long-term storage on the microbiota of a given sample, with the same sequencing technologies and protocols used before and after storage in samples obtained from healthy subjects. Our findings may be applicable only to healthy subjects, as fecal samples from subjects with different fecal consistency (reflecting altered bowel transit), might be more sensitive to long-term storage. This might be the case of patients with diseases, such as inflammatory bowel disease (IBD) although Tedjo et al. did not report difference in gut microbiota structure between healthy and IBD patients, following short-term fecal storage^42^. We aimed here simply to evaluate the effect of the long-term storage of stool samples destined for processing for 16S rRNA gene sequencing, but our findings suggest that long-term storage may influence samples stored for metagenomics, particularly for studies at higher levels of resolution (e.g. at strain level), transcriptomic, metabolomic or proteomic studies^33,43^. Depending on the final use, methods of sample collection and preservation other than those described here may be used, with potentially different impacts on microbiota composition.

## Supplementary information

Supplementary info

## Data Availability

Sequence data associated with this project have been deposited in the NCBI. Short Read Archive under BioProject accession PRJEB23915.
