# Supplementary material for: Effects of the long-term storage of human fecal microbiota samples collected in RNAlater
Source: Sci Rep. 2019 Jan 24;9:601. doi: 10.1038/s41598-018-36953-5 (PMC6345939; doi:10.1038/s41598-018-36953-5)
Supplement: Supplementary file 1 — Supplementary info [file 41598_2018_36953_MOESM1_ESM.docx]

**Effects of the long-term storage of human fecal microbiota samples collected in RNAlater**

Julien Tap^1^, Stéphanie Cools-Portier^1^, Sonia Pavan^2^, Anne Druesne^1^, Lena Öhman^3,4^, Hans Törnblom^4^, Magnus Simren^4,5^, Muriel Derrien^1*^

^1^Danone Nutricia Research, Innovation, Science and Nutrition Department, RD 128 – Avenue de la Vauve, 91767 Palaiseau, France

^2^ SPScienceCom, Paris, France

**^3^** Department of Immunology and Microbiology, Inst. of Biomedicine, University of Gothenburg, Sweden

^4^ Department of Internal Medicine and Clinical Nutrition, Inst. Of Medicine, University of Gothenburg, Sweden

^5^ Center for Functional Gastrointestinal and Motility Disorders, University of North Carolina at Chapel Hill, Chapel Hill, NC, USA.

Running title: Long-term stability of fecal samples

* For correspondence. E‐mail: [muriel.derrien@danone.com](mailto:muriel.derrien@danone.com)

**Supplementary Figure S1. Boxplot of the relative abundance of genera in fecal samples before and after long-term storage.** Genera accounting for more than 5% of the microbiota present in at least one sample are depicted.

**Supplementary Figure S2.** Correlation between the duration of storage in RNAlater (number of days) at room temperature at the subject’s home before stool processing and Bray-Curtis dissimilarity.

**Supplementary Table S1. Number of samples collected in this study**

| Reference dataset | Inter-sequencing run | Fecal storage | Intra-subject | Health status | Study reference |
| --- | --- | --- | --- | --- | --- |
| *N*=24 | - | *N*=24 | - | Healthy subjects | 1 |
| *N*=101 | *N*=19 | - | *N*=93 | IBS subjects | 2 |

**Supplementary Table S2.** Stability of all genera (*N*=99) based on initial prevalence and abundance before long-term storage

|  | Stability (1-BrayCurtis) | mean  rel. abund. | Prevalence (%) |
| --- | --- | --- | --- |
| D_0__Bacteria D_1__Bacteroidetes D_2__Bacteroidia D_3__Bacteroidales D_4__Bacteroidaceae D_5__Bacteroides | 0,959700535 | 0,168318252 | 95,8333333 |
| D_0__Bacteria D_1__Firmicutes D_2__Clostridia D_3__Clostridiales D_4__Lachnospiraceae D_5__Blautia | 0,947470098 | 0,062292624 | 95,8333333 |
| D_0__Bacteria D_1__Firmicutes D_2__Clostridia D_3__Clostridiales D_4__Ruminococcaceae D_5__Faecalibacterium | 0,943768379 | 0,057441126 | 95,8333333 |
| D_0__Bacteria D_1__Firmicutes D_2__Clostridia D_3__Clostridiales D_4__Ruminococcaceae D_5__uncultured | 0,938349757 | 0,030633813 | 95,8333333 |
| D_0__Bacteria D_1__Firmicutes D_2__Clostridia D_3__Clostridiales D_4__Lachnospiraceae D_5__uncultured | 0,921638627 | 0,025174345 | 95,8333333 |
| D_0__Bacteria D_1__Firmicutes D_2__Clostridia D_3__Clostridiales D_4__Lachnospiraceae D_5__Roseburia | 0,914731945 | 0,011368583 | 95,8333333 |
| D_0__Bacteria D_1__Firmicutes D_2__Clostridia D_3__Clostridiales D_4__Ruminococcaceae D_5__Subdoligranulum | 0,912730956 | 0,012356655 | 95,8333333 |
| D_0__Bacteria D_1__Firmicutes D_2__Clostridia D_3__Clostridiales D_4__Lachnospiraceae D_5__Anaerostipes | 0,771948695 | 0,000681452 | 95,8333333 |
| D_0__Bacteria D_1__Firmicutes D_2__Clostridia D_3__Clostridiales D_4__Lachnospiraceae D_5__Pseudobutyrivibrio | 0,938552148 | 0,050273326 | 91,6666667 |
| D_0__Bacteria D_1__Firmicutes D_2__Clostridia D_3__Clostridiales D_4__Lachnospiraceae D_5__Coprococcus | 0,932462644 | 0,012335957 | 91,6666667 |
| D_0__Bacteria D_1__Firmicutes D_2__Clostridia D_3__Clostridiales D_4__Ruminococcaceae D_5__Ruminococcus | 0,917729701 | 0,015966771 | 87,5 |
| D_0__Bacteria D_1__Firmicutes D_2__Clostridia D_3__Clostridiales D_4__Lachnospiraceae D_5__Dorea | 0,800709801 | 0,001992356 | 87,5 |
| D_0__Bacteria D_1__Firmicutes D_2__Clostridia D_3__Clostridiales D_4__Peptostreptococcaceae D_5__uncultured | 0,769499169 | 0,012012457 | 87,5 |
| D_0__Bacteria D_1__Actinobacteria D_2__Actinobacteria D_3__Actinomycetales D_4__Actinomycetaceae D_5__Actinomyces | 0,683022844 | 0,000411945 | 87,5 |
| D_0__Bacteria D_1__Bacteroidetes D_2__Bacteroidia D_3__Bacteroidales D_4__Porphyromonadaceae D_5__Parabacteroides | 0,906072391 | 0,005805328 | 83,3333333 |
| D_0__Bacteria D_1__Firmicutes D_2__Clostridia D_3__Clostridiales D_4__Christensenellaceae D_5__uncultured | 0,888984649 | 0,00827314 | 83,3333333 |
| D_0__Bacteria D_1__Firmicutes D_2__Clostridia D_3__Clostridiales D_4__Defluviitaleaceae D_5__uncultured | 0,737759547 | 0,000734456 | 83,3333333 |
| D_0__Bacteria D_1__Firmicutes D_2__Clostridia D_3__Clostridiales D_4__Ruminococcaceae D_5__Anaerotruncus | 0,735925294 | 0,000460066 | 83,3333333 |
| D_0__Bacteria D_1__Actinobacteria D_2__Actinobacteria D_3__Bifidobacteriales D_4__Bifidobacteriaceae D_5__Bifidobacterium | 0,95610044 | 0,053074495 | 79,1666667 |
| D_0__Bacteria D_1__Bacteroidetes D_2__Bacteroidia D_3__Bacteroidales D_4__Rikenellaceae D_5__Alistipes | 0,916851866 | 0,009772479 | 79,1666667 |
| D_0__Bacteria D_1__Actinobacteria D_2__Coriobacteriia D_3__Coriobacteriales D_4__Coriobacteriaceae D_5__Collinsella | 0,84798137 | 0,01374292 | 79,1666667 |
| D_0__Bacteria D_1__Firmicutes D_2__Clostridia D_3__Clostridiales D_4__Lachnospiraceae D_5__Marvinbryantia | 0,804596594 | 0,001894525 | 79,1666667 |
| D_0__Bacteria D_1__Firmicutes D_2__Bacilli D_3__Lactobacillales D_4__Streptococcaceae D_5__Streptococcus | 0,884572695 | 0,001609157 | 75 |
| D_0__Bacteria D_1__Firmicutes D_2__Erysipelotrichia D_3__Erysipelotrichales D_4__Erysipelotrichaceae D_5__Turicibacter | 0,889246868 | 0,003933236 | 70,8333333 |
| D_0__Bacteria D_1__Proteobacteria D_2__Deltaproteobacteria D_3__Desulfovibrionales D_4__Desulfovibrionaceae D_5__Bilophila | 0,879000059 | 0,001431715 | 66,6666667 |
| D_0__Bacteria D_1__Firmicutes D_2__Erysipelotrichia D_3__Erysipelotrichales D_4__Erysipelotrichaceae D_5__uncultured | 0,655585354 | 0,000656704 | 66,6666667 |
| D_0__Bacteria D_1__Actinobacteria D_2__Coriobacteriia D_3__Coriobacteriales D_4__Coriobacteriaceae D_5__uncultured | 0,877588989 | 0,001160883 | 62,5 |
| D_0__Bacteria D_1__Bacteroidetes D_2__Bacteroidia D_3__Bacteroidales D_4__Porphyromonadaceae D_5__Odoribacter | 0,758509649 | 0,002358488 | 62,5 |
| D_0__Bacteria D_1__Firmicutes D_2__Bacilli D_3__Lactobacillales D_4__Carnobacteriaceae D_5__Granulicatella | 0,690463442 | 0,001327556 | 62,5 |
| D_0__Bacteria D_1__Firmicutes D_2__Clostridia D_3__Clostridiales D_4__Ruminococcaceae D_5__Oscillibacter | 0,614760273 | 0,000276321 | 62,5 |
| D_0__Bacteria D_1__Firmicutes D_2__Negativicutes D_3__Selenomonadales D_4__Veillonellaceae D_5__Dialister | 0,879089314 | 0,006248312 | 58,3333333 |
| D_0__Bacteria D_1__Proteobacteria D_2__Betaproteobacteria D_3__Burkholderiales D_4__Alcaligenaceae D_5__Sutterella | 0,878308575 | 0,002120028 | 58,3333333 |
| D_0__Bacteria D_1__Firmicutes D_2__Clostridia D_3__Clostridiales D_4__Lachnospiraceae D_5__Lachnospira | 0,811863468 | 0,000249764 | 58,3333333 |
| D_0__Bacteria D_1__Proteobacteria D_2__Gammaproteobacteria D_3__Pasteurellales D_4__Pasteurellaceae D_5__Haemophilus | 0,789604045 | 0,000368813 | 58,3333333 |
| D_0__Bacteria D_1__Firmicutes D_2__Negativicutes D_3__Selenomonadales D_4__Acidaminococcaceae D_5__Phascolarctobacterium | 0,816406937 | 0,00086535 | 54,1666667 |
| D_0__Bacteria D_1__Actinobacteria D_2__Coriobacteriia D_3__Coriobacteriales D_4__Coriobacteriaceae D_5__Eggerthella | 0,720432168 | 0,001143432 | 54,1666667 |
| D_0__Bacteria D_1__Firmicutes D_2__Bacilli D_3__Lactobacillales D_4__Lactobacillaceae D_5__Lactobacillus | 0,944178666 | 0,001141908 | 50 |
| D_0__Bacteria D_1__Firmicutes D_2__Clostridia D_3__Clostridiales D_4__Lachnospiraceae D_5__Moryella | 0,924521836 | 0,001597254 | 50 |
| D_0__Bacteria D_1__Firmicutes D_2__Clostridia D_3__Clostridiales D_4__vadinBB60 D_5__uncultured | 0,906013797 | 0,001789115 | 50 |
| D_0__Bacteria D_1__Bacteroidetes D_2__Bacteroidia D_3__Bacteroidales D_4__Prevotellaceae D_5__Prevotella | 0,92095761 | 0,078925361 | 45,8333333 |
| D_0__Bacteria D_1__Actinobacteria D_2__Actinobacteria D_3__Micrococcales D_4__Micrococcaceae D_5__Rothia | 0,461784558 | 0,000131142 | 45,8333333 |
| D_0__Bacteria D_1__Tenericutes D_2__Mollicutes D_3__RF9 D_4__uncultured D_5__uncultured | 0,870102005 | 0,001105177 | 41,6666667 |
| D_0__Bacteria D_1__Bacteroidetes D_2__Bacteroidia D_3__Bacteroidales D_4__Porphyromonadaceae D_5__Butyricimonas | 0,825392588 | 0,000520787 | 41,6666667 |
| D_0__Bacteria D_1__Proteobacteria D_2__Betaproteobacteria D_3__Burkholderiales D_4__Alcaligenaceae D_5__Parasutterella | 0,81169553 | 0,001841122 | 41,6666667 |
| D_0__Bacteria D_1__Bacteroidetes D_2__Bacteroidia D_3__Bacteroidales D_4__Porphyromonadaceae D_5__Barnesiella | 0,841343575 | 0,000966065 | 37,5 |
| D_0__Bacteria D_1__Firmicutes D_2__Negativicutes D_3__Selenomonadales D_4__Veillonellaceae D_5__Veillonella | 0,704183916 | 0,000127149 | 37,5 |
| D_0__Bacteria D_1__Firmicutes D_2__Bacilli D_3__Lactobacillales D_4__Streptococcaceae D_5__Lactococcus | 0,466689461 | 8,69E-05 | 37,5 |
| D_0__Bacteria D_1__Proteobacteria D_2__Deltaproteobacteria D_3__Desulfovibrionales D_4__Desulfovibrionaceae D_5__Desulfovibrio | 0,716649911 | 0,00024902 | 29,1666667 |
| D_0__Bacteria D_1__Firmicutes D_2__Clostridia D_3__Clostridiales D_4__Christensenellaceae D_5__Christensenella | 0,436164053 | 4,25E-05 | 29,1666667 |
| D_0__Bacteria D_1__Firmicutes D_2__Bacilli D_3__Lactobacillales D_4__Leuconostocaceae D_5__Fructobacillus | 0,35531205 | 4,68E-05 | 29,1666667 |
| D_0__Bacteria D_1__Actinobacteria D_2__Actinobacteria D_3__Corynebacteriales D_4__Corynebacteriaceae D_5__Corynebacterium | 0,241565879 | 2,82E-05 | 29,1666667 |
| D_0__Bacteria D_1__Lentisphaerae D_2__Lentisphaeria D_3__Victivallales D_4__Victivallaceae D_5__Victivallis | 0,814649959 | 0,000357488 | 25 |
| D_0__Bacteria D_1__Actinobacteria D_2__Coriobacteriia D_3__Coriobacteriales D_4__Coriobacteriaceae D_5__Enterorhabdus | 0,758388611 | 0,000178794 | 25 |
| D_0__Bacteria D_1__Actinobacteria D_2__Coriobacteriia D_3__Coriobacteriales D_4__Coriobacteriaceae D_5__Slackia | 0,655120802 | 0,000411001 | 25 |
| D_0__Bacteria D_1__Firmicutes D_2__Clostridia D_3__Clostridiales D_4__Lachnospiraceae D_5__Shuttleworthia | 0,63991534 | 7,80E-05 | 25 |
| D_0__Bacteria D_1__Firmicutes D_2__Erysipelotrichia D_3__Erysipelotrichales D_4__Erysipelotrichaceae D_5__Catenibacterium | 0,917076419 | 0,000730157 | 20,8333333 |
| D_0__Bacteria D_1__Firmicutes D_2__Clostridia D_3__Clostridiales D_4__Peptococcaceae D_5__Peptococcus | 0,862852542 | 0,00020234 | 20,8333333 |
| D_0__Bacteria D_1__Proteobacteria D_2__Alphaproteobacteria D_3__Rhodospirillales D_4__Rhodospirillaceae D_5__Thalassospira | 0,848905729 | 0,000556411 | 20,8333333 |
| D_0__Bacteria D_1__Firmicutes D_2__Clostridia D_3__Clostridiales D_4__Peptococcaceae D_5__uncultured | 0,78448311 | 0,000145155 | 20,8333333 |
| D_0__Bacteria D_1__Bacteroidetes D_2__Bacteroidia D_3__Bacteroidales D_4__Prevotellaceae D_5__Paraprevotella | 0,764899908 | 0,001703978 | 20,8333333 |
| D_0__Bacteria D_1__Firmicutes D_2__Clostridia D_3__Clostridiales D_4__Eubacteriaceae D_5__Anaerofustis | 0,573934216 | 3,86E-05 | 20,8333333 |
| D_0__Bacteria D_1__Bacteroidetes D_2__Bacteroidia D_3__Bacteroidales D_4__Porphyromonadaceae D_5__uncultured | 0,500325259 | 4,60E-05 | 20,8333333 |
| D_0__Bacteria D_1__Firmicutes D_2__Clostridia D_3__Clostridiales D_4__Ruminococcaceae D_5__Intestinimonas | 0,359203674 | 3,10E-05 | 20,8333333 |
| D_0__Bacteria D_1__Firmicutes D_2__Erysipelotrichia D_3__Erysipelotrichales D_4__Erysipelotrichaceae D_5__Holdemania | 0,354734269 | 2,06E-05 | 20,8333333 |
| D_0__Bacteria D_1__Actinobacteria D_2__Actinobacteria D_3__Actinomycetales D_4__Actinomycetaceae D_5__Actinobaculum | 0,16429866 | 1,59E-05 | 20,8333333 |
| D_0__Bacteria D_1__Firmicutes D_2__Negativicutes D_3__Selenomonadales D_4__Veillonellaceae D_5__Mitsuokella | 0,902603972 | 0,000915925 | 16,6666667 |
| D_0__Bacteria D_1__Firmicutes D_2__Negativicutes D_3__Selenomonadales D_4__Veillonellaceae D_5__Megasphaera | 0,883730584 | 0,000494889 | 16,6666667 |
| D_0__Bacteria D_1__Firmicutes D_2__Clostridia D_3__Clostridiales D_4__Peptostreptococcaceae D_5__Peptostreptococcus | 0,798780007 | 4,66E-05 | 16,6666667 |
| D_0__Bacteria D_1__Actinobacteria D_2__Coriobacteriia D_3__Coriobacteriales D_4__Coriobacteriaceae D_5__Atopobium | 0,692303716 | 5,38E-05 | 16,6666667 |
| D_0__Bacteria D_1__Firmicutes D_2__Bacilli D_3__Bacillales D_4__Bacillaceae D_5__Bacillus | 0,633379979 | 2,39E-05 | 16,6666667 |
| D_0__Archaea D_1__Euryarchaeota D_2__Methanobacteria D_3__Methanobacteriales D_4__Methanobacteriaceae D_5__Methanobrevibacter | 0,546886502 | 3,59E-05 | 16,6666667 |
| D_0__Bacteria D_1__Proteobacteria D_2__Betaproteobacteria D_3__Burkholderiales D_4__Alcaligenaceae D_5__Achromobacter | 0 | 1,27E-05 | 16,6666667 |
| D_0__Bacteria D_1__Fusobacteria D_2__Fusobacteriia D_3__Fusobacteriales D_4__Fusobacteriaceae D_5__Fusobacterium | 0,725966356 | 0,000108714 | 12,5 |
| D_0__Bacteria D_1__Firmicutes D_2__Clostridia D_3__Clostridiales D_4__Ruminococcaceae D_5__Flavonifractor | 0,707823462 | 2,85E-05 | 12,5 |
| D_0__Bacteria D_1__Firmicutes D_2__Clostridia D_3__Clostridiales D_4__Lachnospiraceae D_5__Acetitomaculum | 0,587453744 | 4,89E-05 | 12,5 |
| D_0__Bacteria D_1__Firmicutes D_2__Clostridia D_3__Clostridiales D_4__Ruminococcaceae D_5__Hydrogenoanaerobacterium | 0,561950577 | 2,81E-05 | 12,5 |
| D_0__Bacteria D_1__Actinobacteria D_2__Actinobacteria D_3__Propionibacteriales D_4__Propionibacteriaceae D_5__Propionibacterium | 0,406858254 | 2,75E-05 | 12,5 |
| D_0__Bacteria D_1__Actinobacteria D_2__Coriobacteriia D_3__Coriobacteriales D_4__Coriobacteriaceae D_5__Gordonibacter | 0,349437388 | 5,99E-05 | 12,5 |
| D_0__Bacteria D_1__Firmicutes D_2__Bacilli D_3__Bacillales D_4__Staphylococcaceae D_5__Staphylococcus | 0,282194806 | 1,94E-05 | 12,5 |
| D_0__Bacteria D_1__Proteobacteria D_2__Epsilonproteobacteria D_3__Campylobacterales D_4__Campylobacteraceae D_5__Campylobacter | 0 | 1,59E-05 | 12,5 |
| D_0__Bacteria D_1__Bacteroidetes D_2__Bacteroidia D_3__Bacteroidales D_4__Prevotellaceae D_5__uncultured | 0,978379713 | 0,000417973 | 8,33333333 |
| D_0__Bacteria D_1__Firmicutes D_2__Clostridia D_3__Clostridiales D_4__Lachnospiraceae D_5__Butyrivibrio | 0,923029285 | 6,52E-05 | 8,33333333 |
| D_0__Bacteria D_1__Tenericutes D_2__Mollicutes D_3__Anaeroplasmatales D_4__Anaeroplasmataceae D_5__Anaeroplasma | 0,701276714 | 0,00042292 | 8,33333333 |
| D_0__Bacteria D_1__Firmicutes D_2__Clostridia D_3__Clostridiales D_4__Lachnospiraceae D_5__Howardella | 0,671386562 | 4,20E-05 | 8,33333333 |
| D_0__Bacteria D_1__Actinobacteria D_2__Coriobacteriia D_3__Coriobacteriales D_4__Coriobacteriaceae D_5__Olsenella | 0,659920728 | 1,27E-05 | 8,33333333 |
| D_0__Bacteria D_1__Bacteroidetes D_2__Bacteroidia D_3__Bacteroidales D_4__Porphyromonadaceae D_5__Coprobacter | 0,646790936 | 4,52E-05 | 8,33333333 |
| D_0__Bacteria D_1__Firmicutes D_2__Clostridia D_3__Thermoanaerobacterales D_4__Thermoanaerobacteraceae D_5__Gelria | 0,25033347 | 7,27E-06 | 8,33333333 |
| D_0__Bacteria D_1__Actinobacteria D_2__Actinobacteria D_3__Actinomycetales D_4__Actinomycetaceae D_5__Varibaculum | 0 | 1,83E-05 | 8,33333333 |
| D_0__Bacteria D_1__Firmicutes D_2__Clostridia D_3__Clostridiales D_4__Lachnospiraceae D_5__Oribacterium | 0 | 8,41E-06 | 8,33333333 |
| D_0__Bacteria D_1__Bacteroidetes D_2__Bacteroidia D_3__Bacteroidales D_4__Prevotellaceae D_5__Alloprevotella | 0,96277526 | 0,001321739 | 4,16666667 |
| D_0__Bacteria D_1__Firmicutes D_2__Erysipelotrichia D_3__Erysipelotrichales D_4__Erysipelotrichaceae D_5__Solobacterium | 0,925434498 | 3,02E-05 | 4,16666667 |
| D_0__Bacteria D_1__Bacteroidetes D_2__Bacteroidia D_3__Bacteroidales D_4__uncultured D_5__uncultured | 0,801192191 | 2,49E-05 | 4,16666667 |
| D_0__Bacteria D_1__Bacteroidetes D_2__Bacteroidia D_3__Bacteroidales D_4__Porphyromonadaceae D_5__Porphyromonas | 0,691168358 | 2,13E-05 | 4,16666667 |
| D_0__Bacteria D_1__Firmicutes D_2__Bacilli D_3__Lactobacillales D_4__Leuconostocaceae D_5__Weissella | 0,433071142 | 2,43E-06 | 4,16666667 |
| D_0__Bacteria D_1__Firmicutes D_2__Erysipelotrichia D_3__Erysipelotrichales D_4__Erysipelotrichaceae D_5__Asteroleplasma | 0,417592633 | 1,21E-05 | 4,16666667 |
| D_0__Bacteria D_1__Actinobacteria D_2__Actinobacteria D_3__Actinomycetales D_4__Actinomycetaceae D_5__Mobiluncus | 0 | 9,51E-06 | 4,16666667 |
| D_0__Bacteria D_1__Firmicutes D_2__Clostridia D_3__Clostridiales D_4__Eubacteriaceae D_5__Eubacterium | 0 | 6,71E-06 | 4,16666667 |
| D_0__Bacteria D_1__Firmicutes D_2__Clostridia D_3__Clostridiales D_4__Ruminococcaceae D_5__Saccharofermentans | 0 | 2,89E-06 | 4,16666667 |
| D_0__Bacteria D_1__Firmicutes D_2__Negativicutes D_3__Selenomonadales D_4__Acidaminococcaceae D_5__Acidaminococcus | 0 | 7,10E-06 | 4,16666667 |

**Table S3.** Overview of studies comparing the effect of different stool collection procedures and/or the storage of fecal samples on the composition of the microbiota

| **Aim of the study** | **Number of subjects tested and characteristics of fecal sample donors** | **Standard** | **Process of collection of fecal samples before storage at -80°C** | **Stability at -80°C measured** | **Analytical method, Sequencing technology** | **Results summary** | **Reference** | **Considered for Figure 6** |
| --- | --- | --- | --- | --- | --- | --- | --- | --- |
| Impact of long term storage at -80°C | N= 24 | DNA extracted before long-term storage | RNAlater (at RT) 12 ± 5 days | Yes (5 years) | 16S rRNA sequencing (V5-V6 region) 454 platform | Microbiota variability lower than intra-subject variability. Some genera are more affected than others | This study | yes |
| Impact of collection procedures and long term storage at -80°C | N=4 | RT for 30 min | RT for 30 min , 1, 4, 6, 8 or 24h, RT for 30 min, -80°C for 1wk, 1, 2, 3, 4, 5 or 6 mo | Yes, 1wk, 1, 2, 3, 4, 5 or 6 mo | 16S rRNA sequencing (V1-V3 region),454 platform | The composition of the microbiota in fecal samples stored at RT (up to 24h) or -80°C (up to 6 mo) clustered strongly based on the host each sample originated from, No influence of length of storage and temperature on microbiota composition, Exception for 1 IBS subject: variable microbiota composition (phylum level) between RT and -80°C storage; lower stability at RT than healthy subjects | 3 | yes |
| Impact of short term storage at -80°C | N=7 | Fresh (within 4h of defecation) | Snap frozen in dry ice, then -80°C for 7d, -80°C for 7 days, Fresh stool. | Yes (7days) | 16S rRNA sequencing (V3-V4 region), Illumina MiSeq, Culture methods | No significant differences at phylum or family levels between treatment conditions, At genus level, effect of snap freezing (vs fresh) on *Faecalibacterium* and *Leuconostoc*, Samples clustered based on subject, rather than by storage group | 4 | yes |
| Impact of long term storage at -20°C | n=40 | Freshly processed samples from a different study cohort with similar demographic and health characteristics | Freeze-dried, then -20°C for ~14 years | No (-20°C) | 16S rRNA sequencing, (V3-V4 region), MiSeq platform | Higher DNA shearing in long-term stored samples (but still compatible with amplicon-based studies), At phylum level, similar composition as in the compared cohort, and similar weighted and unweighted UniFrac distances | 5 | no |
| Impact of collection procedures and short term storage at -80°C | N=2 | -80°C | Storage at 20°C, 4°C, -20°C or -80°C for 3 or 7 days | Yes (3 and 7 days) | 16S rRNA sequencing (V1-V2 region) 454 platform | Phylogenetic structure and diversity of communities in individual samples were not significantly influenced by temperature or duration of storage, relative abundances of most taxa (phylum to order level) were largely unaffected by temperature even after 14 days of storage | 6 | yes |
| Impact of long term storage at -80°C | N=9 |  | 4h to 5 weeks at RT, then -80°C for up to 2 years | Yes (2 years) | 16S rRNA sequencing,(V4 region), MiSeq platform, | Number of OTUs decreases with time at -80°C(stool tested only from 4 premature infants) , Change in abundance of some genera over time during -80°C storage (Increase lactobacilli, decrease Staphylococci), Alpha-diversity for storage at RT (Shannon Index) decreases over time with premature infants, After 1 wk, stability decrease, Less complex diversity (premature infants) was more strongly affected by loss and gain of low abundance OTUs. Increased *Bifidobacteria*, decreased *Veillonella*, | 7 | yes |
| Impact of collection procedures | N=7 | Anaerobic storage at -20°C | anaerobic storage at -20°C , 4-10°C or RT for 1 week in RNAlater, 4°C for 24h in RNAlater, | No | Whole genome shotgun sequencing | Clustering was not affected by the storage method, No abundance difference between storage methods (species level), Between-subjects variability is higher than storage effect, Decreased Shannon diversity index with samples stored at RT, or 4-10°C for 1wk in RNA*Later*® compared to fresh frozen samples (not with samples stored for 24h) | 8 | yes |
| Impact of collection procedures | N=10 | -80°C | -80°C, ice for 24 or 48h, 48h at RT in stool stabilizer, then -80°C, immediate processing (3 extra samples) | No | Different DNA extraction kits,16S rRNA sequencing,(V1-V2 region),454 platform,(GS FLX and Titanium) | Limited effect of storage method on major taxa, Variation between individuals higher than variation due to storage or extraction method, Limited effect of 454 GS FLX sequencing versus Titanium,“Storage times at -80°C ranged from 0-137 days; time of storage at -80°C had no discernable effect on the sequencing results.”, | 9 | yes |
| Impact of collection procedures | N=16 | Fresh | Fresh, -20°C, OMNIgene-gut stabilization, Storage 28 days | No | WGS, HiSeq platform | No difference between storage conditions for abundance, diversity, functional profiles, Intra-subject variation greater than storage method variation | 10 | no |
| Impact of collection procedures | N=4 |  | RT for 3h, 24h, 48h, 72h or 14 d, -20°C in home freezer, -20°C in home freezer, thawing for 1h or 3h, RT in RNAlater for 3h, 24h, 14 days or 1 month | No | 16S rRNA sequencing (V4 region),454 platform | Decreased DNA and RNA integrity with longer storage >24h at RT and freeze-thawing. High heterogeneity with use of RNA Later (added outside laboratory), Number of total species not affected, Composition at genus and species level is affected by prolonged storage at RT (14d) and by thawing-refreezing, Intra-individual variability > inter-individual variability | 11 | yes |
| Impact of collection procedures | N=1 | -80°C | -80°C, 4°C, Tris-EDTA buffer at RT, RNAlater at RT, OMNIgeneGut at RT, no additive at RT, Storage for 72h |  | 16S rRNA sequencing (V4 region), Illumina MiSeq platform | samples stored using other conditions showed substantial divergence, compared to −80 °C control samples, Aside from refrigeration, the use of OMNIgene.GUT resulted in the least alteration, while the greatest change was seen in samples stored in TE buffer, | 12 | no |
| Impact of collection procedures | N=3 | -80°C | FOBT, RT no additive, RNAlater, -80°C, Storage for 3 days | Yes (3 days) | 16S rRNA sequencing, (V3-V4 region) 454 platform | Overall microbiome structure differed by subject but microbiome structure and relative abundance of major taxa (phyla) did not differ by collection method. No difference in Shannon Index (diversity) between methods except with RNA*Later* | 13 | yes |
| Impact of collection procedures | N=10 | Snap freezing in dry ice | no media or RNAlater or RNAlater + kanamycin or RNAlater + ciprofloxacin, 3 days or 7 days at 25°C, Snap freezing in dry ice | No | 16S rRNA sequencing (V3-V4 region), Illumina MiSeq platform | Microbiota composition is stable for 7 days at RT only when using RNA*Later*®, Without medium, microbiota composition is severely affected, Analysis of rare taxa inaccurate when samples are not frozen immediately | 14 | yes |
| Impact of collection procedures | N=8 | Fresh frozen on dry ice, | fresh frozen on dry ice, ethanol for 48h at RT, RNAlater for 48h at RT | No | Shotgun sequencing, Illumina HiSeq | Within-subject microbial species, gene, and transcript abundances were highly concordant across sampling methods, with only a small fraction of transcripts (<5%) displaying between-method variation. | 15 | yes |
| Impact of collection procedures | N=7 |  | -80°C within 15 min of defecation, one stool: fresh subsampling of inside and outside, one stool: homogenized in liquid N_2_ (powder), 15-30 min at RT, one stool: homogenized in liquid N_2_, subsamples stored in home freezer for 0, 3, 7, 14, 30 days, -80°C storage ± RNAlater, |  | 16S rRNA, qPCR | Homogenization reduces taxa variability, Storage at RT for 30 min alters the abundance of major taxa. Freezing within 15 min is recommended, Storage in home freezer alters the abundance of taxa | 16 | no |
| Impact of collection procedures | N=29 | Fresh sample | immediately at -80°C, 15 min, 30 min, 1h or 2h at RT | No | 16S rRNA sequencing, (V3-V5 region), 454 Titanium platform | Overall compositional and structural differences of microbial communities were due to inter-individual differences, Alpha diversity did not differ between samples with storage time from 0 to 2 hours, More changes in relative abundance of specific taxa after 1 or 2h (genus level) (increase of aerobes and facultative anaerobes), | 17 | no |
| Impact of collection procedures | N=42 | Fresh | Fresh, OMNIgene Gut with stabilizer for 1 or 2 weeks | No | 16S rRNA sequencing, (V4-V5 region), MiSeq platform | no significant effect of storage on the abundance (genus level) and diversity in either infants or elderly samples (due to variance between subjects), but infant samples tended to display greater difference in composition between freshly extracted and stored samples, when aggregating infant and elderly samples, changes in relative abundance of *Faecalibacterium*, *Sporobacter*, *Clostridium* XVIII, and *Clostridium* XlVa after 1 wk storage, and also of *Bacteroides* after 2wk, | 18 | no |
| Impact of collection procedures | N=4, |  | Parallel analyses in 2 labs, Fecal samples kept at 4°C, processed within 4h | No | DNA extraction with MoBio Powersoil or MP Biomedicals FastDNA SPIN Kit, 16S rRNA sequencing, (V3-V5 region), 454 Titanium platform | Important differences in DNA yield, samples extracted with MoBio Powersoil showed increased *Bacteroidaceae*, *Ruminococcaceae* and *Porphyromonadaceae*, and lower *Enterobacteriaceae*, *Lachnospiraceae*, *Clostridiaceae*, and *Erysipelotrichaceae*, Differences in relative abundance between labs was limited (only for *Sutterellaceae)*, | 19 | no |
| Impact of collection procedures | N=10 | Snap freezing in liquid N_2_ | drying (Whatman cards, silica gel beads), 5 days at RT, RNAlater, 5 days at RT, PAXgene, 5 days at RT, 4°C for 24h, | No | Different DNA extraction kits, qPCR 16s rRNA *Bacteroides spp.* | Variable amounts of total DNA and of *Bacteroides* depending on extraction method and treatment of fecal sample (higher yield with RNA*Later*® and Qiagen QIAamp DNA Stool Mini), Holding samples for up to 5d in preservation solution is compatible with DNA extraction and qPCR, | 20 | no |
| Impact of collection procedures | N=5 | Snap freezing in liquid N_2_ | Snap freezing in liquid N_2_, 8 or 24h at 4°C or RT, | No | SSCP (V4-V5 region) and ABI PRISM sequencing, qPCR 16s rRNA | Bacterial diversity and total number of bacteria significantly reduced after 8 and 24 h at both RT and 4°C | 21 | no |
| Impact of collection procedures | N=4 | -80°C | -80°C , 12, 24, 48, 72h at RT | No | 16S rRNA sequencing, (V3 region), 454 platform | high inter-individual variability in diversity and abundance (higher than storage effect), changes in diversity increase with time at RT (little change at 12h), Abundance of some taxa (genus level) changed with time of storage at RT | 22 | no |
| Impact of collection procedures | N=20 |  | no additive, RNAlater, 70% ethanol, EDTA, dry swab, FOBT, For each condition, -80°C or RT for 24 or 96h, then -80°C, Parallel analyses in 2 labs | no | 16S rRNA sequencing (V4 region or V3-V5), Illumina MiSeq platform | Microbial variability was due primarily to individual differences, followed by sampling method, then by storage time, effect of storage at RT (4d vs 1d) on relative phyla abundances and alpha diversity metrics, FOBT and RNA*later* resulted in the highest stability without freezing for four days (low stability with ethanol), With FOBS and no additive, some OTUs displayed pronounced growth after 4d at RT, swab, FOBT, and 70% ethanol exhibited the greatest accuracy when immediately frozen | 23 | no |
| Impact of collection procedures | N=10 | Processing within 2-5h | no additive, 70% or 95% ethanol, RNAlater, OMNIgene Gut, FTA cards. For each condition, -20°C, 4°C, RT, freeze-thawing cycles or fluctuating temps (4-40°C), Processing within 2-5h or after 1, 4 or 8 weeks | No | 16S rRNA sequencing (V4 region), Illumina Hiseq and Illumina MiSeq platforms | 95% ethanol, FTA cards, and the OMNIgene Gut kit can preserve samples sufficiently well at ambient temperatures such that differences at 8 weeks are comparable to differences among technical replicates, with no additive or 70% ethanol, changes in microbial communities were comparable in effect size to differences between individuals, storage method has greater effect on abundance than diversity, Changes in abundance (OTUs) varied across taxonomic groups, depending on which stabilizer was used, with FTA cards showing the smallest fluctuations | 24 | no |
| Impact of collection procedures | N=28 | -80°C | -80°C, -20°C for 1 week, then -80°C, 4°C or RT for 24h, then -80°C, Fecal transport swabs 48-72h at RT, then -80°C | No | 16S rRNA sequencing, (V1-V3 region), 454 platform,qPCR 16s rRNA *Methanobrevibacter smithii* | Alpha diversity did not differ between storage methods and -80°C, except for the fecal swabs (genus level), clustering by test subject but not by storage method, no effect of the storage methods in the subgroups of healthy subjects, IBS and IBD patients, no effect of storage method on the presence of specific bacterial taxa, and of the relative abundance of *Faecalibacterium*,and *Roseburia* (except for fecal swabs samples) | 25 | no |
|  |  |  |  |  |  |  |  |  |
| N, number of individuals; SSCP, single-stranded conformation polymorphism; OTU, operational taxonomic unit; IBS, irritable bowel syndrome; IBD, inflammatory bowel disease; FOBT: fecal occult blood test, RT: Room temperature. | | | | | | | | |

**References**

1 Tillisch, K. *et al.* Consumption of Fermented Milk Product With Probiotic Modulates Brain Activity. *Gastroenterology* **144**, 1394-1401.e1394, doi:<http://dx.doi.org/10.1053/j.gastro.2013.02.043> (2013).

2 Tap, J. *et al.* Identification of an Intestinal Microbiota Signature Associated With Severity of Irritable Bowel Syndrome. *Gastroenterology* **152**, 111-123.e118, doi:<http://dx.doi.org/10.1053/j.gastro.2016.09.049> (2017).

3 Carroll, I. M., Ringel-Kulka, T., Siddle, J. P., Klaenhammer, T. R. & Ringel, Y. Characterization of the fecal microbiota using high-throughput sequencing reveals a stable microbial community during storage. *PLoS One* **7**, e46953, doi:10.1371/journal.pone.0046953 (2012).

4 Fouhy, F. *et al.* The effects of freezing on faecal microbiota as determined using MiSeq sequencing and culture-based investigations. *PLoS One* **10**, e0119355, doi:10.1371/journal.pone.0119355 (2015).

5 Kia, E. *et al.* Integrity of the human faecal microbiota following long-term sample storage. *PLoS One* **11**, e0163666, doi:10.1371/journal.pone.0163666 (2016).

6 Lauber, C. L., Zhou, N., Gordon, J. I., Knight, R. & Fierer, N. Effect of storage conditions on the assessment of bacterial community structure in soil and human-associated samples. *FEMS Microbiol Lett* **307**, 80-86, doi:10.1111/j.1574-6968.2010.01965.x (2010).

7 Shaw, A. G. *et al.* Latitude in sample handling and storage for infant faecal microbiota studies: the elephant in the room? *Microbiome* **4**, 40, doi:10.1186/s40168-016-0186-x (2016).

8 Voigt, A. Y. *et al.* Temporal and technical variability of human gut metagenomes. *Genome Biol* **16**, 73, doi:10.1186/s13059-015-0639-8 (2015).

9 Wu, G. D. *et al.* Sampling and pyrosequencing methods for characterizing bacterial communities in the human gut using 16S sequence tags. *BMC Microbiol* **10**, 206, doi:10.1186/1471-2180-10-206 (2010).

10 Andersson, A. F. *et al.* Comparative analysis of human gut microbiota by barcoded pyrosequencing. *PLOS ONE* **3**, e2836, doi:10.1371/journal.pone.0002836 (2008).

11 Cardona, S. *et al.* Storage conditions of intestinal microbiota matter in metagenomic analysis. *BMC Microbiol* **12**, 158, doi:10.1186/1471-2180-12-158 (2012).

12 Choo, J. M., Leong, L. E. & Rogers, G. B. Sample storage conditions significantly influence faecal microbiome profiles. *Sci Rep* **5**, 16350, doi:10.1038/srep16350 (2015).

13 Dominianni, C., Wu, J., Hayes, R. B. & Ahn, J. Comparison of methods for fecal microbiome biospecimen collection. *BMC Microbiol* **14**, 103, doi:10.1186/1471-2180-14-103 (2014).

14 Flores, R. *et al.* Collection media and delayed freezing effects on microbial composition of human stool. *Microbiome* **3**, 33, doi:10.1186/s40168-015-0092-7 (2015).

15 Franzosa, E. A. *et al.* Relating the metatranscriptome and metagenome of the human gut. *Proc Natl Acad Sci U S A* **111**, E2329-2338, doi:10.1073/pnas.1319284111 (2014).

16 Gorzelak, M. A. *et al.* Methods for improving human gut microbiome data by reducing variability through sample processing and storage of stool. *PLoS One* **10**, e0134802, doi:10.1371/journal.pone.0134802 (2015).

17 Guo, Y. *et al.* Effect of short-term room temperature storage on the microbial community in infant fecal samples. *Sci Rep* **6**, 26648, doi:10.1038/srep26648 (2016).

18 Hill, C. J. *et al.* Effect of room temperature transport vials on DNA quality and phylogenetic composition of faecal microbiota of elderly adults and infants. *Microbiome* **4**, 19, doi:10.1186/s40168-016-0164-3 (2016).

19 Kennedy, N. A. *et al.* The impact of different DNA extraction kits and laboratories upon the assessment of human gut microbiota composition by 16S rRNA gene sequencing. *PLoS One* **9**, e88982, doi:10.1371/journal.pone.0088982 (2014).

20 Nechvatal, J. M. *et al.* Fecal collection, ambient preservation, and DNA extraction for PCR amplification of bacterial and human markers from human feces. *J Microbiol Methods* **72**, 124-132, doi:10.1016/j.mimet.2007.11.007 (2008).

21 Ott, S. J. *et al.* In vitro alterations of intestinal bacterial microbiota in fecal samples during storage. *Diagn Microbiol Infect Dis* **50**, 237-245, doi:10.1016/j.diagmicrobio.2004.08.012 (2004).

22 Roesch, L. F. *et al.* Influence of fecal sample storage on bacterial community diversity. *Open Microbiol J* **3**, 40-46, doi:10.2174/1874285800903010040 (2009).

23 Sinha, R. *et al.* Collecting Fecal Samples for Microbiome Analyses in Epidemiology Studies. *Cancer Epidemiol Biomarkers Prev* **25**, 407-416, doi:10.1158/1055-9965.epi-15-0951 (2016).

24 Song, S. J. *et al.* Preservation Methods Differ in Fecal Microbiome Stability, Affecting Suitability for Field Studies. *mSystems* **1**, doi:10.1128/mSystems.00021-16 (2016).

25 Tedjo, D. I. *et al.* The effect of sampling and storage on the fecal microbiota composition in healthy and diseased subjects. *PLoS One* **10**, e0126685, doi:10.1371/journal.pone.0126685 (2015).
